# Updates on Post-Resuscitation Care. After the Return of Spontaneous Circulation beyond the 2021 Guidelines

**DOI:** 10.31083/j.rcm2311373

**Published:** 2022-10-31

**Authors:** Alessandro Fasolino, Sara Compagnoni, Enrico Baldi, Guido Tavazzi, Johannes Grand, Costanza N.J. Colombo, Francesca Romana Gentile, Luca Vicini Scajola, Federico Quilico, Clara Lopiano, Roberto Primi, Sara Bendotti, Alessia Currao, Simone Savastano

**Affiliations:** ^1^Division of Cardiology, Fondazione IRCCS Policlinico San Matteo, 27100 Pavia, Italy; ^2^Department of Molecular Medicine, University of Pavia, 27100 Pavia, Italy; ^3^Department of Medical, Surgical, Diagnostic and Pediatric Science, University of Pavia, 27100 Pavia, Italy; ^4^Anesthesiology and Intensive Care Unit, Fondazione IRCCS Policlinico San Matteo, Fondazione IRCCS Policlinico San Matteo, 27100 Pavia, Italy; ^5^Department of Cardiology Copenhagen University Hospital, Hvidovre and Amager-Hospital, 2650 Copenhagen, Denmark

**Keywords:** cardiac arrest, ROSC, post-ROSC care, resuscitation

## Abstract

Out-of-hospital cardiac arrest is one of the leading causes of mortality worldwide. The goal of resuscitation is often meant as the return of spontaneous circulation (ROSC). However, ROSC is only one of the steps towards survival. The post-ROSC phase is still a challenging one during which the risk of death is all but averted. Morbidity and mortality are exceedingly high due to cardiovascular and neurologic issues; for this reason, post ROSC care relies on international guidelines, the latest being published on April 2021. Since then, several studies have become available covering a variety of topics of crucial importance for post-resuscitation care such as the interpretation of the post-ROSC ECG, the timing of coronary angiography, the role of complete myocardial revascularization and targeted temperature management. This narrative review focuses on these new evidences, in order to further improve clinical practice, and on the need for a multidisciplinary and integrated system of care.

## 1. Introduction

Out-of-hospital cardiac arrest (OHCA) is one of the leading cause of mortality in industrialized countries, with and incidence of 56/100,000 per year of started resuscitations in Europe [1] and 74.3/100,000 per year in the US [2]. Following the Utstein formula of survival [3] great effort has been deployed into the identification and treatment of this medical emergency by acting on medical science, local implementation and system improving. However, while the return of spontaneous circulation (ROSC) is obtained in about a third of all the cases with resuscitation attempted, only 8% of them survive to hospital discharge [1]. Mortality remains high after ROSC because of the occurrence of post-cardiac-arrest syndrome, characterized by brain injury, myocardial dysfunction, systemic ischaemia/reperfusion response and the persistent precipitating pathology. Post-cardiac-arrest syndrome is the major determinant of death after ROSC with a bimodal distribution of predominant causes: in the early phase (emergency department admission) mortality is mainly driven by cardiovascular causes (i.e., hemodynamic instability, recurrent arrest, intractable shock), whereas in the later phases (in-hospital admission) mortality is mainly due to neurological issues [neurological withdrawal of life-sustaining therapy (WLST) and brain death] [4]. To improve the quality of care in such a delicate situation post-ROSC guidelines are periodically published [5]. However, since the publication of the latest guidelines, new evidence has become available covering several pivotal issues of post-ROSC care such as coronary artery revascularization and targeted temperature management (TTM). The purpose of this review is to underline the relevant updates in post-resuscitation science tracing a modern and integrated system of post-ROSC care.

## 2. ECG Acquisition and Interpretation

As suggested by both the European and the American guidelines [5, 6] the very first decisional step after ROSC is grounded on the acquisition of a 12-lead electrocardiogram (ECG). Post-ROSC ECG is aimed to drive the first crucial decision namely to decide whether an immediate coronary angiography (CAG) is needed. As discussed more in detail in the next section, this indication is reserved for those patients with a presumptive diagnosis of ST-segment elevation myocardial infarction (STEMI). From here it comes that the right interpretation of ECG leads to the right decision.

Nevertheless, its interpretation is not as straightforward as in the stable patients with typical chest pain and may resent from several limitations which could affect the sensitivity and the specificity of ECG for STEMI diagnosis.

In cardiac arrest patients we have to deal with several issues, namely the global ischemia owed to the no-flow or the low-flow phase during cardiac arrest and the persistence of hemodynamic instability after ROSC, both of whom might decrease the diagnostic accuracy of standard ECG. Thus, in order to improve the treatment of post-ROSC patients, it is imperative to correctly recognize false negative or false positive ECGs for STEMI also in this peculiar setting.

**False negatives**: in case of acute transmural myocardial infarction the ST segment is not always clearly elevated; this is particularly true in case of left main coronary artery or proximal anterior interventricular artery thrombosis and in case of severe multivessel disease [7]. In these circumstances the decision to perform an immediate CAG must rely on signs of ongoing ischemia, hemodynamic and/or electrical instability, echocardiographic signs of regional wall motion abnormalities and serial ECG evaluation. False negatives’ reduction is important to reduce the delay of a CAG, which can improve the outcome of patients with myocardial infarction.

**False positives**: the issue of falsely positive ECGs for STEMI is often a challenge for clinicians. The inappropriate activations of the cath lab are not uncommon [8] and many cardiac and non-cardiac causes other than coronary occlusion may be associated with such an ECG pattern [9]. To further complicate ECG interpretation in this critical post-ROSC period non-coronary transmural myocardial ischemia, commonly due to global and subsequently myocardial ischemia during the no-flow and low-flow phases of cardiac arrest, results in a pathological ST elevation even in the absence of significant coronary artery obstructions. These ischaemic electrocardiographic signs may disappear after restoration of good systemic perfusion as well as they may persist in case of persisting hypoperfusion.

**Fig. 1.**
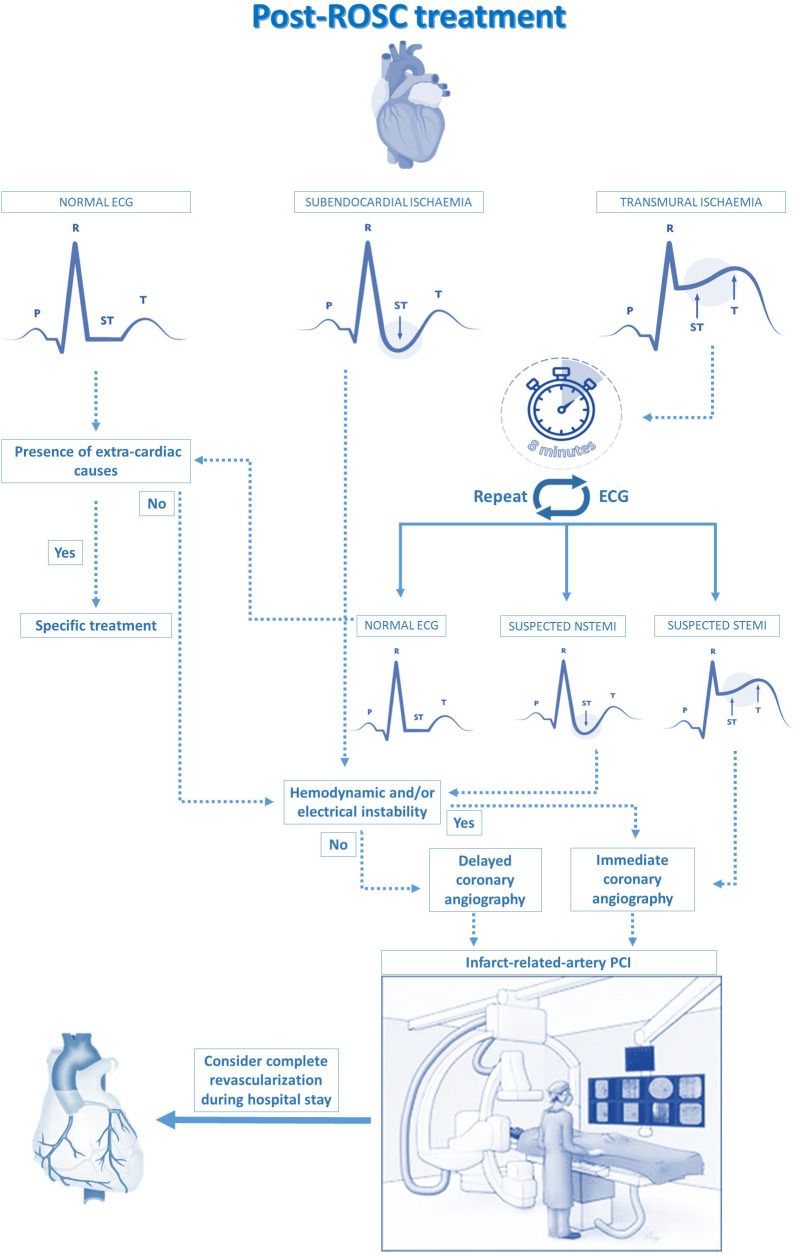
**A proposed algorithm for post-ROSC treatment: the cardiologist’s point of view**. This panel focuses on the role of ECG and its interpretation, on the timing of CAG and on the indication to a complete myocardial revascularization.

In a study from our group [10] the ECGs performed in the first 7 minutes after ROSC had higher rate of false positive for STEMI (18.5%), as compared to those performed between 7–33 minutes (7.2%) or after 33 minutes (5.8%). Hence, the simple acquisition, or the repetition, of the ECG after 8 minutes from ROSC halves the rate of false-positive ECG for STEMI (Fig. 1). On the other hand, global hypoperfusion may continue after ROSC because of post-arrest myocardial stunning, systemic ischaemia/reperfusion response or the persistence of the precipitating disease and this can be disclosed by indicators of poor perfusion such as the peripheral perfusion index (PI). New evidence from our group suggests that prolonged low values of peripheral perfusion index lasting for up to 30 minutes after ROSC (measured with standard peripheral pulse-oximetry devices with a mean value obtained in the 30 minutes after ROSC – MPI30) negatively affect the reliability of ECG [11]. In fact, when MPI30 was lower than 1 the rate of false-positive ECG was about 30% whereas if MPI30 was higher than 2.6 the rate of false-positive ECG falls to less than 4%. Not surprisingly prolonged hypoperfusion with persistency of low MPI30 values was associated also with worse outcomes [12]. The reduction of the false positive rate is aimed to reduce the possible complications of unnecessary CAG in patients with extracardiac causes of cardiac arrest (i.e., aortic dissection or intracranial bleeding). For all these reasons our suggestion is to abandon the old paradigm of acquiring the 12-lead ECG as soon as possible after ROSC rather at the right time and to implement the hemodynamic condition of the patients into the ECG interpretation.

## 3. CAG Indications

The majority of sudden cardiac deaths among adults are due to arrhythmias secondary to cardiac ischemia [13], which is why CAG is one of the pivotal diagnostic steps for OHCA survivors. As stated before, the main indication for an immediate CAG is the presence of ST segment elevation [5, 14]. This indication is based on the proven high prevalence of a culprit lesion in patients with ST segment elevation, or left bundle branch block, after ROSC [15, 16] and on the beneficial effect of early revascularization [17, 18, 19]. Moreover, while there is no doubt about the beneficial role of an urgent invasive approach for STEMI patients, now it is also clear that this approach does not improve survival of patients without persistent ST segment elevation. Two randomized controlled trials, published in the New England Journal of Medicine in 2019 and 2021 [20, 21] clearly showed that an immediate CAG was not beneficial over a delayed strategy with a follow up of 90 and 30 days respectively. Even though not statistically significant, in both trials the survival rate of the immediate angiography group was slightly lower than that of the delayed group. These results could be explained by potentially harmful consequences of an invasive approach: first of all, the non-recognition of a non-cardiac etiology of cardiac arrest (i.e., sub-arachnoid hemorrhage with secondary alteration of the ST-segment), leading to a delayed treatment of the primary cause and exposing the patient to procedural risks, unnecessary anticoagulant or antiplatelet therapy [22]. The results of these two trials are not to be confused with those of a previous study [19] highlighting how immediate coronary intervention was associated with a better survival both in STEMI and non-STEMI patients. The crux of the matter is that in this latter study [19] they compared survival of patients according to whether they received or not an immediate coronary angioplasty, but all the patients considered in this study underwent urgent CAG. This is quite expected because in the presence of a culprit lesion a coronary angioplasty is supposed to be beneficial. The two studies of 2019 and 2021, instead, compared immediate versus delayed CAG. Once again, we have to leave the old way directing immediately to the cath lab every patient resuscitated from cardiac arrest, without evident extra-cardiac causes, and we should follow a more modern approach selecting for immediate CAG only patients with STEMI. There could be the need for an immediate CAG also for patients with myocardial infarction without ST-segment elevation (NSTEMI) but, according to latest guidelines, the only indication is the presence of ongoing hemodynamic and/or electrical instability [5, 23].

## 4. Revascularization Strategy in Coronary Multivessel Disease

It is common knowledge that patients suffering from an acute coronary syndrome (complicated or not by OHCA) benefit from primary coronary intervention (p-PCI) of the infarct-related-artery (IRA) in terms of survival. However, significant multivessel obstructive coronary artery disease is common in these patients, as well as in the subset of OHCA patients, ranging from 25 to 50% [20, 21, 24]. Complete revascularization of non-IRA lesions and the relative timing (whether during the index hospitalization or electively, after patient’s stabilization and discharge) remain a matter of debate in all the following different populations. Concerning NSTEMI patients, guidelines recommend multivessel revascularization during the index hospitalization, with a preference on multi-stage (IIa) versus single-stage (IIb) procedures [23]. However, clinical studies have shown superiority [25], or at least non-inferiority [26], of the single-stage approach. For STEMI patients there is a class IIa recommendation for complete treatment of multivessel disease during the index hospitalization [14]. For the subset of patients with ischemic cardiogenic shock (CS) the guidelines diverge with a recommendation on immediate complete revascularization for STEMI (IIa) versus a recommendation against routine complete revascularization for NSTEMI patients complicated by CS (III). This is probably due to the publication in 2017 of the CULPRIT-SHOCK trial [27], which showed superiority of the revascularization of the IRA alone in the acute setting, with an option of complete revascularization in a staged procedure (17.7% of the culprit-only arm went on to perform this procedure), versus complete revascularization in the acute setting of CS. When it comes to patients after cardiac arrest there are no recommendations available on revascularization strategy in case of coronary multivessel disease. A recent retrospective study from our group [28] suggested that survival with good neurologic outcome of patients who received a complete revascularization, either during the index or staged procedure during the index hospitalization, was higher than the IRA-only group (83.3% vs. 30.4%, *p *< 0.001). Even after correction for renal function, cardiac arrest duration, shockable rhythm, and the need for a pharmacologic or mechanical circulatory support a complete revascularization was confirmed to be independently associated with survival [HR 3 (95% CI 1.1–10), *p* = 0.04]. Another retrospective study [29] showed that immediate complete versus incomplete revascularization of three-vessel disease or left main coronary artery was associated with higher neurologically intact survival, even though the follow up was limited to one month. Further randomized studies for the subpopulation of OHCA patients presenting with multivessel coronary artery disease are needed to further clarify the best revascularization strategy. Based on the current and available evidence, we suggest proceeding to complete revascularization during the index hospitalization (Fig. 1).

## 5. Post-ROSC Echocardiography

Guidelines recommend to perform echocardiography “as soon as possible” after ROSC in order to detect any persistent precipitating cardiac pathology and to quantify the degree of myocardial dysfunction [5, 30]. This practice is supported by the evidence that, at least in patients suffering from cardiac arrest of cardiac etiology, performing 2D echocardiography within 24 hours from the event is associated with higher survival [31]. Echocardiography can also be used as a non-invasive way to monitor hemodynamic variables such as cardiac index. Even though invasive CI measurements along the first 24 hour after cardiac arrest in patients undergoing TTM was not associated with mortality in patients with normal lactate levels [32], a non-invasive monitoring might be useful for tailoring the treatment. Efforts have been made to identify strong echocardiographic predictors of outcomes after cardiac arrest, but no echocardiographic parameter of left or right ventricular, systolic or diastolic, function has been consistently and independently associated with survival [33, 34]. The only exception was an isovolumic relaxation time (IVRT) >100 ms, which was shown to be independently associated [35] with poor survival [HR 3.3 (95% CI 1.6–6.7), *p* = 0.002]. Perhaps, more than focusing on a single examination, the real value of echocardiographic assessment after cardiac arrest lies on the possibility to perform a serial assessment, in order to identify the trend towards improvement. For example, a study showed [36], that the increase of both cardiac index and left ventricular ejection fraction (LVEF) was associated with higher survival. Further studies in this field are probably forthcoming, with the implementation of newer technologies such as speckle-tracking imaging and strain analysis, limited at the moment to animal models in experimental settings [37].

## 6. Targeted Temperature Management

For comatose patients after cardiac arrest, the ERC-ESICM guidelines recommend monitoring core temperature and preventing fever (temperature >37.7 °C) for at least 72 hours [38]. Fever prevention can be accomplished by administering anti-pyretic drugs, uncovering of the patient, or by using cooling devices. Cooling or temperature control at 32–36 °C is not recommended any more after cardiac arrest. Temperature control (initially termed as therapeutic hypothermia) with a target for core temperature of 32–34 °C was the first neuroprotective intervention. It was introduced in the international guidelines in 2002 [39, 40, 41] based on two small RCTs, of which the first was the “Hypothermia after cardiac arrest (HACA)-trial” published in 2002 [42]. In this trial, 273 resuscitated patients after OHCA with ventricular fibrillation were randomized to temperature control with a target temperature of 32–34 °C for 24 hours or standard therapy. The trial was stopped prematurely due to lack of funding. After 6 months, mortality was 41% in the hypothermia group and 55% in the control group (risk ratio 0.74, 95% CI 0.58–0.95). In the same year, results from a smaller quasi-randomized trial of 77 patients supported the HACA-trials results [43]. The intervention was identical in terms of temperature-target, but duration was shorter (12 hours). Mortality in-hospital was 51% in the intervention group and 68% in the control group. These results soon led to intensive research within therapeutic hypothermia in various settings and timings. Several trials investigated whether rapid initiation of hypothermia, started in the prehospital setting, would improve outcomes [44, 45, 46, 47]. Both hypothermia during resuscitation such as intra-arrest cooling [44, 45, 47] and hypothermia immediately after return of spontaneous circulation was investigated, but no benefit was seen from these approaches [44, 46, 48]. Because of some concerns were raised regarding the methodology in risk of bias in the two initial trials of therapeutic hypothermia [49], a large trial of almost 1000 patients was initiated to evaluate the effect of therapeutic hypothermia (now termed as targeted temperature management, TTM). In the TTM1-trial, 939 patients were randomly allocated to 33 °C or 36 °C for 24 hours [50]. Fever was treated for the first 72 hours in both groups. The trial found no difference in mortality or neurological function after 6 months between the two groups. In the following years, guidelines have allowed for a target temperature of 36 °C and 33 °C, but temperature target should remain constant during TTM [5]. In 2019, 584 comatose survivors of cardiac arrest (mix of in-hospital and out-of-hospital) due to non-shockable rhythm (asystole or pulseless electrical activity) were included in the HYPERION trial [51]. This trial showed a significantly higher survival with good neurological outcome in the intervention group compared with normothermia. However, in 2021 the TTM-2 trial reported no difference in survival with good neurological outcome at 6 months among 1850 comatose OHCA-patients [52]. Patients were included irrespective of initial rhythm. In this trial, the intervention consisted as previous trials in targeting a core temperature at 33 °C for 24 hours and subsequently, preventing fever for a total of 72 hours. The control group was only treated if patients developed fever, defined as body temperature >37.7 °C.

## 7. Oxygenation and Ventilation

Refractory cardiac arrest and/or comatose patients after ROSC always require ventilation to both protect the airways and control the homeostasis of blood gas analysis. The oxygenation target has been a matter of debate for many years and the optimal PaO2 levels were unknown; evidence suggests there is a U-shaped relationship between PaO2 levels with higher mortality and worsening functional status at the extremes [53]. Significant hypoxemia may further aggravate the altered DO2/VO2 relation worsening end-organ perfusion, which is usually already compromised by the cardiac dysfunction in cardiac arrest patients. Hyperoxemia may lead to cellular damage related to the production of reactive oxygen species [53, 54]. A recent pooled analysis from two trials, with small sample size, compared low vs high oxygen therapy (100% oxygen compared to a lesser amount that was titrated by using a pulse oximeter) in the prehospital setting with no significant association between low oxygen therapy and survival to hospital discharge [55].

Recently the BOX trial (a superiority multicentric interventional randomized clinical trial with 2 × 2 factorial design allocating comatose OHCA patients to one of the two target blood pressures and to an open blind restrictive (9–10 kPa; 68–75 mmHg) vs. liberal (13–14 kPa; 98–105 mmHg) oxygenation therapy in comatose out-of-hospital cardiac arrest patients) has been published [56]. The study randomized 789 patients resulted in a similar incidence of death or severe disability or coma between the 2 groups (respectively at 90 days: 126 of 394 patients—32.0%—of whom 113 died in the restrictive-target group and in 134 of 395 patients—33.9%—of whom 113 died in the liberal-target group [adjusted HR 0.95; 95% confidence interval 0.75–1.21; *p* = 0.69]). Of note, the median time of ROSC was 21 minutes with a high proportion presenting with shockable rhythms, rapid initiation of CPR and higher values of PaO2 than the target limits planned in the study.

**Fig. 2.**
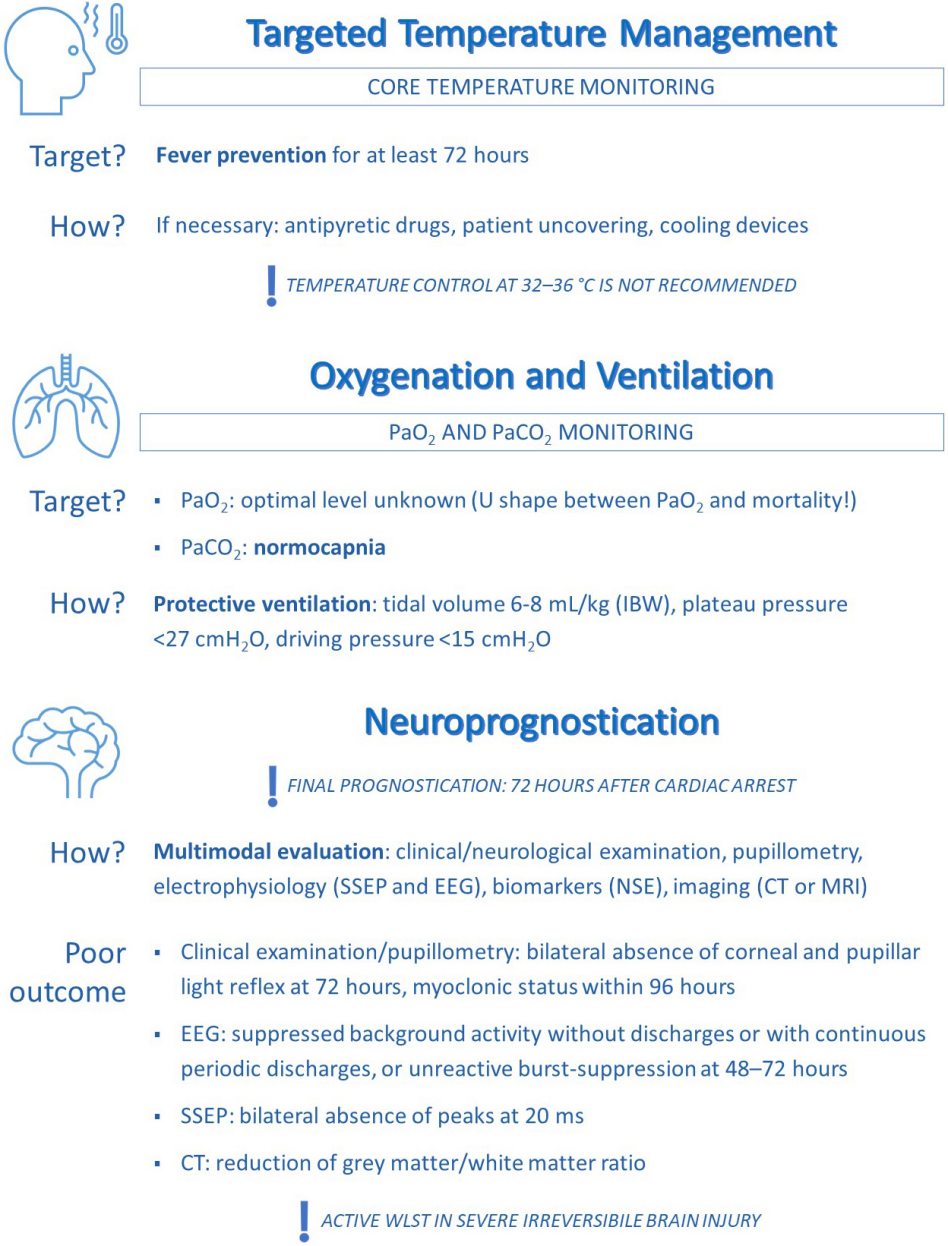
**A proposed algorithm for post-ROSC treatment: the intensivists’ point of view**. This panel focuses on temperature management, ventilation and neuroprognostication.

Without evidence suggesting precise values of PaCO2, normocapnia is suggested to avoid detrimental effect on cerebral circulation and pressure. Observational data suggest that patients undergoing TTM are prone to hypocapnia, therefore a strict monitoring of carbon dioxide with arterial blood gas analysis and the use of end tidal CO2 monitoring should be routinely performed with the titration of ventilation parameters to achieve the desirable range of values. Almost all the studies on the ventilation and oxygenation in comatose post-cardiac arrest patients focus mainly on the role of the gas analysis rather than on ventilation modes. In a secondary analysis of three prospective, observational multicenter studies including 812 patients from 1998 to 2010, demonstrated that a significant reduction in tidal volume, peak and plateau pressure, and a significant increase of respiratory rate and PEEP were observed over the years [57]. A recent secondary analysis of the TTM2 trial showed that respiratory rate, driving pressure (plateau pressure - PEEP), mechanical power and ventilatory ratio are independently associated with 6-month mortality with the formula [(4 × Driving Pressure) + RR] being also associated with mortality and poor neurological outcome [58]. However, mechanical power and ventilatory ratio are not universally applied as part of the daily routine at bedside. In the lack of specific evidence, the application of protective ventilation by using a tidal volume of 6–8 mL/kg (ideal body weight) and avoiding high airway pressure (Plateau pressure <27 cmH2O and driving pressure <15 cmH2O) by titrating pressure control and PEEP in pressure ventilation modalities and tidal volume and PEEP in volume ventilation modes in order to achieve normoxia and normo-carbia is suggested. The same applies when extracorporeal circulatory support is onsite (Fig. 2).

## 8. Neuroprognostication

In patients with ROSC after OHCA, who are admitted to hospital in a comatose state, the mortality is as high as 50% and most deaths are from hypoxic-ischemic brain injury [59]. Active WLST is commenced in the patients with severe irreversible brain injury; however, it can be difficult to distinguish this patient-group from patients with a potential for late recovery [60]. Accurate prognostication is extremely important to avoid prolongation of the suffering of patients and relatives and to avoid inappropriate WLST. No single parameter or test can certainly predict the prognosis. Therefore, international guidelines recommend the use of multiple tests and clinical observations in a multimodal prognostication model to guide clinicians [5]. The 2015 ERC-ESICM Guidelines on Post-Resuscitation Care proposed a model for the prediction of poor neurological outcome for comatose patients after cardiac arrest [41]. Retrospective studies have validated this model [61]. The prognostication model is based on a combination of tests including results of clinical/neurological examination, electrophysiology (Short-latency somatosensory evoked potentials - SSEP, Electroencephalogram - EEG), biomarkers (neuron specific enolase - NSE), and imaging (CT or MRI). Bilateral absence of both corneal and pupillary light reflexes at 72 hours predicts poor outcome with high specificity but low sensitivity. Automated quantitative pupillometry has been shown to be superior to manual pupillometry for predicting neurological outcome and it is recommended in recent guidelines [5, 62, 63]. Status myoclonus within 96 hours is associated with poor outcome, but in these patients an EEG is important to characterize the phenotype of the myoclonus since some patients survive despite myoclonus with good outcome [64, 65]. A highly malignant EEG involving suppressed background activity without discharges or with continuous periodic discharges, or unreactive burst-suppression at 48–72 hours indicates poor outcome [66]. Also, EEG without malignant signs predicts good outcome. An important confounder is sedation, which may influence EEG-patterns. SSEP with bilateral absence of peaks at 20 ms (known as N20 signals) is close to 100% specific of a poor prognosis, but with low sensitivity [5]. Blood-borne biomarkers, such as neuron-specific enolase, are associated with brain damage and poor neurological outcome [67]. Cutoff values, however, vary between studies and it has proved difficult to perfectly distinguish survivors form non-survivors. Head CT can be indicated as initial diagnostics for potential intracranial hemorrhage as a cause of the arrest. For neuroprognostication, the reduction of the grey matter/white matter ratio on brain CT within 72 hours after ROSC is useful when combined with other prognosticators of poor neurologic outcome in comatose patients after OHCA [5]. Measurement of the grey matter/white matter ratio expressed in Hounsfield units is a method to assess the degree of cerebral oedema. This ratio is normally higher than 1, meaning that grey matter has the highest density. Lower ratio is worse and associated with greater degree of brain injury [68]. Final prognostication should not be decided until at least 72 hours after OHCA. As no features are perfect predictors of outcome, the multimodal prognostication model in addition to cautious expectation is essential for the management of survivors of cardiac arrest remaining comatose (Fig. 2).

## 9. The Role of a Multidisciplinary Approach: The Cardiac Arrest Centres

As largely discussed in the previous sections, post-resuscitation care is grounded on a series of interventions provided by different healthcare providers. Cardiologists, for the indication and timing of the CAG and for differential diagnosis of causative underlying cardiac pathologies; interventional cardiologists for coronary intervention and/or to position percutaneous left ventricle assist devices; intensivists, for early post-ROSC care, temperature management, ventilation and prognostication; cardiac surgeons, for surgical myocardial revascularization or in case an extracorporeal membrane oxygenator is needed; cardiac electrophysiologists for catheter ablation and/or for ICD implantation. All these actions are essential for survival and are more likely to be provided in high volume hospitals. A study published in 2012 showed a better survival in post-ROSC patients admitted in high volume hospitals as compared to low-volume ones and this was confirmed also in the different subgroups according to cardiac arrest etiology [69]. The authors concluded that “*This analysis is relevant to regionalized cardiac arrest care systems that include a designated high volume cardiac resuscitation center and supporting EMS systems*” strengthening what was already suggested by a policy statement of the American Heart Association in 2010 [70]. This document went beyond the definition of high-volume hospitals as clearly enumerated the criteria that a regional centre should have to receive post-ROSC patients. These hospitals will be called cardiac arrest centres (CAC) in the following years both in the US [71] and in Europe [72, 73]. A recent study from Korea [74] on a vast sample of patients (more than 95,000) showed that the direct transport to a CAC was associated with an increased survival of about two times. Interestingly, at least for patients with a shockable presenting rhythm, the benefit in survival was independent of the time needed to reach the CAC, meaning that a longer transport but to a CAC was preferable to a shorter transfer but to a non-CAC [non-CAC <8 min; non-CAC >8 min: OR 0.40 (95% CI 0.12–1.32); CAC <8 min: OR 1.92 (95% CI 1.26–2.94); CAC >8 min: OR 1.78 (95% CI 1.03–3.10)] [75]. The reason of these findings lays on the presence at CAC of a multidisciplinary team able to provide all the procedures and the diagnostic support that patients need to increase their chance of survival.

A multidisciplinary approach is, in fact, strongly recommended also by the latest guidelines on ventricular arrhythmias and prevention of sudden death by the European Society of Cardiology [30].

## 10. Conclusions

The achieving of the return of spontaneous circulation is not only the goal of resuscitation but the beginning of a challenging journey characterized by high mortality due to cardio-circulatory causes and neurological ones. During such a delicate phase it is of pivotal importance to put into practice a multidisciplinary approach which involves cardiologists, interventional cardiologists, intensivists, cardiac surgeons and cardiac electrophysiologists (if needed) providing the patients with the best tailored treatment in order to enhance survival.

## Abbreviations

CAC, Cardiac arrest centres; CAG, coronary angiography; CPR, Cardiopulmonary resuscitation; CS, Cardiogenic shock; CT, Computed tomography; DO2, Oxygen delivery; ECG, 12-lead electrocardiogram; EEG, Electroencephalogram; ICD, Implantable cardioverter-defibrillator; IRA, infarct-related-artery; IVRT, Isovolumic relaxation time; LVEF, Left ventricular ejection fraction; MPI30, Mean value of perfusion index obtained in the 30 minutes after return of spontaneous circulation; MRI, Magnetic resonance imaging; NSE, Neuron specific enolase; NSTEMI, Myocardial infarction without ST-segment elevation; OHCA, Out-of-hospital cardiac arrest; PCI, Percutaneous coronary intervention; PEEP, Positive end-expiratory pressure; PI, Perfusion index; ROSC, Return of spontaneous circulation; SSEP, Short-latency somatosensory evoked potentials; STEMI, ST-segment elevation myocardial infarction; TTM, Targeted temperature management; VO2, Oxygen consumption; WLST, withdrawal of life-sustaing therapy.
